# Sweet basil oil nanoparticles as novel feed additives: impacts on productivity, intestinal morphology, antioxidant capacity, and microbiota in laying hens

**DOI:** 10.5713/ab.260348

**Published:** 2026-09-01

**Authors:** Suphasuta Tada, Sureerat Thuekeaw

**Affiliations:** 1 Faculty of Animal Science and Technology, Maejo University, Chiang Mai, Thailand

**Keywords:** Antioxidant Capacity, Egg Production, Intestinal Targeted Delivery, Laying Hen, Lipid Peroxidation, Nanocapsule

## Abstract

**Objective:**

Nanotechnology has been suggested as a promising strategy for enhancing the stability and delivery efficiency of feed additives through the formation of nanoparticles (NPs). Sweet basil oil (SBO) contains a diverse range of bioactive constituents; however, its intestinal-targeted delivery remains limited. Therefore, this study aimed to determine the effects of sweet basil oil nanoparticles (SBO-NP) on egg production and egg quality, intestinal morphology, antioxidant capacity and cecal microbiota in laying hens.

**Methods:**

A total of 225 Lohmann Brown laying hens at 25 wk of age were randomly assigned to three treatments with five replicates of 15 birds in a completely randomized design. Dietary treatments were as follows: (i) basal diet (Con), (ii) basal diet with free SBO at 500 ppm, (iii) basal diet with SBO NPs at 500 ppm (SBO-NP), respectively. The experiment was conducted for 10 wk, during which egg production and quality, antioxidant capacity, intestinal morphology, and cecal microbiota were assessed.

**Results:**

Dietary SBO-NP increased egg production than SBO and Con diets (p<0.01). However, all additives had no effect on feed conversion ratio and egg quality. The SBO-NP markedly improved villus height and villus surface area (p<0.05). Furthermore, superoxide dismutase activity in duodenum and liver increased by SBO-NP. Malondialdehyde levels were reduced by 52%–53% in the SBO-NP group compared to the Con group (p<0.05). In contrast, the cecal microbiota remained broadly similar among treatments at the phylum and genus levels with no significant differences in the alpha and beta diversity.

**Conclusion:**

The results demonstrated that the SBO-NPs enhanced egg production by improving intestinal morphology, increasing antioxidant capacity, and reducing lipid peroxidation.

## INTRODUCTION

The egg industry has shown strong growth over the past decade. By 2034, the United States Department of Agriculture (USDA) projects an 8.2% increase in egg consumption compared to 2024, driven by demand for protein sources [1]. However, the egg production continues to face significant challenges in ensuring the health and productivity of laying hens. In parallel, commercial layer production is affected by multiple stressors, including climate change, high stocking density, and pathogens exposure. These factors disrupt redox homeostasis, gut barrier integrity, microbiota balance, thereby reducing laying performance, egg quality and causing economic losses [2]. Therefore, promoting antioxidant capacity, preserving the intestinal architecture and understanding of intestinal microbiota colonization patterns in laying hens are essential for maintaining the health and productivity of laying hens.

The antioxidant defense systems play a crucial role in maintaining reproductive efficiency and egg production in laying hens. Because it helps reduce free radicals that damage cell membranes, intestines, liver, and ovaries crucial for egg production, especially under heat stress conditions [3,4]. A well-developed intestinal architecture, characterized by an increased surface area for the absorption of nutrients such as amino acids, calcium (Ca), and phosphorus (P), supports efficient nutrient utilization, thereby enhancing egg, albumen, and eggshell production in laying hens [5]. In addition, the intestinal microbiota is closely associated with the host’s physiological status, and egg production can be improved through modulation of the gut–reproductive axis microbiome, which influences not only intestinal function but also reproductive tract health and metabolic processes [6,7].

As outlined above, these challenges highlight the necessity for innovative feed additives capable of addressing sustainability, efficiency, and animal health. It is widely acknowledged that dietary plant-derived bioactive compounds have been utilized for enhancing egg production and its quality, regulating oxidative balance, and supporting immune function [8]. Sweet basil oil (SBO; *Ocimum basilicum*), belonging to the *Lamiaceae* family, can be used as a phytogenic feed additive for poultry due to its phytochemical constituents. The SBO contains main active compounds such as estragole (methyl chavicol), linalool and 1,8-cineole [9]. These compounds exhibit antioxidant properties, inhibit pathogenic microorganisms [10] and stimulate the secretion of digestive enzymes, resulting in improved gut integrity and nutrient utilization [11]. Studies have shown that administering a diet with 0.05% basil oil improved production performance and antioxidant capacity in broilers [12]. Feeding basil oil alleviated the oxidative stress by increasing superoxide dismutase (SOD) in rats challenged by iron nanoparticles (NPs) [13]. Additionally, dietary supplementation with basil oil at 400 and 800 mg/kg reduced malondialdehyde (MDA) levels in meat and increased *Lactobacillus* populations in the cecal content [14].

However, maintaining the active compounds and their effectiveness in the gastrointestinal (GI) tract is limited due to easy deterioration, low bioavailability and sensitivity to unfavorable conditions. To protect poorly absorbed and easily degraded compounds, nanotechnology has been suggested as a possible strategy to form NPs for controlled release of substances at the targeted site, thereby enhancing their stability [15]. The NPs also have the ability to cross biological barriers more effectively and prolong intestinal residence, owing to their biocompatibility, high surface area, reduce the required dosage of feed additives [16]. Thus, additive delivery systems based on nanotechnology contributes to improved productivity and animal health in livestock farming [17]. Previously, nanoencapsulated pomegranate peel by alginate retained a higher viable probiotic cell than free cells after exposure to the simulated gastric juice [18]. Thymol nanoencapsulated in sodium alginate (SA) mitigated the negative effects of *Salmonella* Typhimurium challenge on growth performance in broilers [19]. Among natural polymers, alginate and chitosan (CS) are biodegradable and biocompatible polymers. The complex structure of alginate and CS provides a protective barrier against environmental factors and controls the release of encapsulated compounds [20].

Nanoencapsulated phytogenic compounds have demonstrated several beneficial effects in poultry. For instance, nanoencapsulated silymarin mitigated oxidative stress associated with hepatotoxicity in broiler chickens [21]. Nanoencapsulated garlic essential oil improved growth performance in broiler chickens [22]. Similarly, the nanoencapsulated form of lavender essential oil enhanced nutrient digestibility and improved intestinal morphology in broiler chickens [23]. Despite the increasing interest in phytogenic additives and nanotechnology in poultry, most available studies have focused on broilers, while limited information is available on the use of basil-oil-based NPs in laying hens, particularly regarding their effects on intestinal morphology, antioxidant capacity, the gut microbiome, egg production, and egg quality. Therefore, this study aimed to assess the efficacy of dietary SBO nanocapsules on the responses of intestinal morphology, antioxidant status as well as cecal microbiota in laying hens. Egg production and quality were also evaluated.

## MATERIALS AND METHODS

### Feed additive preparation

Sweet basil oil was purchased from commercially available products (Thai-China Flavours and Fragrances Industry). The active ingredients of SBO were quantified using gas chromatography-mass spectrometry, following the method of Qasem et al. [24] with minor modifications. In brief, the sample was diluted 20-fold in hexane. The mixture (0.2 μL) was injected into the GC system at a split temperature of 250°C. The bioactive constituents were identified using their retention time and were expressed as percentages. Sweet basil oil nanoparticles (SBO-NP) were prepared by encapsulating SBO within the SA-CS matrix and were used as the feed additive in the present study.

### Total phenolic contents and 2,2-diphenyl-1-picrylhydrazyl radical scavenging activity

Total phenolic content was evaluated using the method described by Caponio et al. [25]. Briefly, the reaction mixture consisted of 500 μL of sample, 2,000 μL of Folin–Ciocalteu reagent, and 1,000 μL of sodium carbonate solution. The mixture was incubated in the dark at room temperature for 2 h. The absorbance was then measured at 765 nm using a Biochrom Libra S50 UV-Vis spectrophotometer. A gallic acid calibration curve was prepared at concentrations ranging from 20 to 200 μg/mL. The results were expressed as milligrams of gallic acid equivalent to mL of sample (mg GAE/mL extract). Antioxidant activity was determined using the 2,2-diphenyl-1-picrylhydrazyl (DPPH) assay, according to the method of Brand-Williams et al. [26] with some modifications. Briefly, 400 μL of either SBO (0.2 g/mL) or SBO-NP (0.2 g/mL) was mixed with 2,000 μL of DPPH solution (0.2 mM in methanol) and incubated in the dark for 30 min. The absorbance was measured at 517 nm. Trolox was used as the positive control for these analyses.

### Animal care

The study was conducted from May to November in 2025 at the Faculty of Animal Science and Technology, Maejo University, Chiang Mai, Thailand.

### Animals, diets, and experimental design

A total of 225 Lohmann Brown laying hens were initially obtained at 18 weeks of age (WOA) from a local farm. The hens were fed a basal diet for 7 wk, during which egg production increased to 50% of the flock. During the adaptation period, laying hens aged 24 WOA were weighed (initial body weight: 1.6±0.1 kg) and randomly allocated into three groups (75 hens/group), with five replicates (15 hens/pen) in a completely randomized design. Hens were temporarily leg-tagged for body weight identification before being randomly allocated to treatment groups, with 5 replicates per treatment. All procedures were conducted by trained personnel who were blinded to the treatment assignments. The collection period began at 25 WOA. The dietary treatments consisted of (i) basal diet (Con), (ii) basal diet with SBO at 500 ppm and (iii) basal diet with SBO NPs at 500 ppm, respectively. The inclusion level of 500 ppm was selected based on our previous study [27]. The experiment was conducted for 10 wk. All experimental diets were formulated to meet or exceed the nutrient requirement based on National Research Council [28]. The composition of the diet is presented in Table 1. The hens were housed at a density of 1.6 m^2^ per bird in a conventional house, with free access to feed and water. The light was provided for 16 h throughout the entire experimental period.

### Egg production and egg quality

Laying performance was assessed by measuring average daily feed intake (ADFI) and feed-to-egg ratio (FCR) on a weekly basis. Egg number and egg weight were recorded daily at a consistent time and used to calculate average egg weight (AEW) and hen-day egg production (HD, %). On the last 3 d of each 5-wk interval, egg quality was conducted by collecting a total of 90 eggs (30 eggs per treatment) to measure eggshell strength (kg/m^2^), eggshell thickness (mm), albumen height (mm), yolk color, yolk index, yolk height (mm) and Haugh unit (HU). All parameters were measured using a digital egg tester (DET6500; NABEL).

### Intestinal histomorphology

At 34 WOA, three birds from each replicate were randomly selected and euthanized by CO_2_ inhalation. Approximately 2 to 3 cm segments were collected from the midpoint of the duodenum, jejunum, and ileum. These samples were collected in 10% buffered formalin, fixed in paraffin, placed on glass slides, and stained with hematoxylin and eosin. Intestinal histomorphology was analyzed by measuring villus height (VH), villus width (VW) and crypt depth (CD) at 40x magnification (Olympus, Mode BX5). Then, villus height-to-crypt depth ratio (VH:CD) and villus surface area (VSA) were subsequently calculated from the measured VH, VW, and CD values. The 3 values obtained within each replicate were averaged before statistical analysis. The replicate mean (n = 5) was used as the experimental unit. ImageJ and EPview software were used for measurement and image analysis.

### Antioxidant capacity

For analyzing redox status including total antioxidant capacity (T-AOC), SOD, glutathione peroxidase (GSH-Px), and catalase (CAT) and MDA, the liver and duodenal mucosa were collected from 3 birds within each replicate and pooled to obtain one representative biological sample. Consequently, each treatment consisted of 5 pooled biological replicates (n = 5). The duodenal mucosa was gently scraped with a sterile glass slide. The samples were homogenized in cold phosphate-buffered saline and centrifuged at 3,000×g for 10 min at 4°C. The supernatant was carefully collected for further analysis.

The T-AOC was determined using the 2,2′-azino-bis (3-ethylbenzothiazoline-6-sulfonic acid) (ABTS) assay based on the reduction of the ABTS radical cation [29]. The SOD activity was assessed by observing the changes in chemiluminescence of luminol. The activity of GSH-Px was measured using the colorimetric method described by Habig et al. [30]. The CAT activity was determined by monitoring the decomposition of H_2_O_2_ at 240 nm [31]. The thiobarbituric acid reactive substances (TBARS) method was used to indicate the MDA level by preparing a thiobarbituric acid (TBA) solution mixed with the sample (1:1 ratio) and then heating at 100°C for 30 min [32].

### Cecum microbiota

For cecum microflora analysis, cecal digesta from 3 birds within each replicate were aseptically collected into one sample per replicate, immediately kept in liquid nitrogen, and stored at −80°C until further analysis. Total genomic DNA was extracted from the cecal contents using the High Pure PCR Template Preparation Kit (Roche) according to the manufacturer’s protocol. The concentration and quality of the extracted DNA were assessed using a Qubit fluorometer (Thermo Fisher Scientific). Samples were then barcoded, and the PCR products were quantified and purified before sequencing. Sequencing output files were generated in POD5 format, converted to FASTQ format, and subsequently used for downstream microbiome analysis. Bacterial community composition was determined by 16S rRNA gene sequence data obtained from the Oxford Nanopore Technologies platform. The taxonomic classification was then performed using the EPI2ME workflow wf-16s version 1.4.0, with minimap2 version 2.28-r1209 used for alignment with the NCBI 16S/18S targeted-loci reference database. The minimum criteria for accepting classification results are a sequence similarity of at least 95% and reference sequence coverage of at least 90%. No separate denoising or explicit chimera-detection/removal step was applied. Downstream filtering, normalization, and statistical analyses were performed in Microbiome Analyst using the taxonomic abundance table generated from EPI2ME. Microbial diversity was assessed using alpha diversity indices (Shannon and Chao1) and beta diversity was assessed using the Bray–Curtis dissimilarity index at the feature level. Principal coordinate analysis (PCoA) was performed to visualize differences in microbial community composition among the experimental groups, and statistical significance was evaluated using PERMANOVA with pairwise comparisons. Biomarker identification was performed using Random Forest classification and linear discriminant analysis effect size (LEfSe) across multiple taxonomic levels. The microbiome sequencing data have been submitted to the NCBI Sequence Read Archive (SRA) and are available under BioProject accession number PRJNA1497593.

### Statistical analysis

All data were analyzed using the one-way analysis of variance (ANOVA) procedure in SAS software (SAS Institute). The replicate pen was considered as the experimental unit. Mean values were compared using post-hoc Tukey’s HSD test with statistical significance considered at p≤0.05. Results were presented as mean and standard error of the mean (SEM). Statistical analysis was performed with the following model:


(1) $${\text{Y}}_{ij}=\mu +{\text{T}}_{i}+{\text{e}}_{ij}$$

where Y*_ij_* is the dependent variable observation, μ is the overall mean, T*_i_* is the effect of the treatment, and e*_ij_* is the random error.

## RESULTS

### Bioactive compounds, total phenolic content and antioxidant activity

Analysis of the active compounds in SBO is illustrated in Table 2. Estragole (methyl chavicol) was the most abundant active compound, accounting for 77.51%, followed by linalool (7.75%), trans-alpha-bergamotene (2.90%), *β*-ocimene (2.38%), eucalyptol (1.15%), and other active compounds (~8.31%). The SBO and SBO-NPs samples had EC_50_ of 1.29±0.01 mg/mL and 1.26±0.01 mg/mL, respectively.

### Influence of dietary sweet basil oil-nanoparticles on antioxidant capacity in liver and duodenum

The antioxidant status was assessed using the following indicators: T-AOC, SOD, CAT, GSH-Px activities, and MDA content (Table 3). T-AOC in the duodenum ranged from 0.41–0.79 μmol, whereas T-AOC was slightly decreased in the liver (0.33–0.40 μmol). Laying hens fed SBO-NPs had the highest T-AOC in the duodenum, which was different from the SBO and Con groups (p<0.01). No difference was observed between SBO-NPs and SBO groups on T-AOC in the liver; however, both groups differed from Con group (p = 0.022). Dietary treatments did not affect the activities of SOD, CAT, and GSH-Px in the duodenal mucosa. Similarly, no differences were found in SOD and GSH-Px activities in the liver. In contrast, hepatic CAT enzyme activity increased in laying hens receiving SBO-NPs supplement as comparable to the Con (p<0.01). In addition, hens fed the diet supplemented with SBO-NPs showed a higher activity than those fed the SBO diet (p<0.01). The highest of MDA level was observed in both the duodenum (6.27 nmol/mL) and liver (5.07 nmol/mL) of laying hen receiving the Con diet. Interestingly, the dietary addition of SBO reduced MDA levels by 35.4% in the duodenum and 20.7% in the liver, while MDA levels in the duodenum and liver reduced, when supplementing the diet with SBO-NPs by 52%–53% compared to the Con group.

### Influence of dietary sweet basil oil-nanoparticle on intestinal histomorphology

The intestinal morphology of laying hens at 34 WOA is shown in Table 4. In duodenal part, SBO-NP influenced VH and CD (p<0.01) compared to those fed the Con and SBO diets. SBO-NPs also increased VW than Con diet (p = 0.01), but no difference was found between the SBO and SBO-NPs groups. The VH:CD was unaffected by the diets. Conversely, the supplementation with SBO-NPs resulted in the greatest increase in VSA (p<0.01). The morphology of the jejunum showed similar trends to the duodenum. Hens fed the Con diet had the lowest VH (938.53 μm). Comparing the two groups, the diet supplemented with SBO-NPs showed a better increase in VH than the diet supplemented with SBO (p<0.01), but no difference in VW was found. The VH:CD (p = 0.039) and VSA (p<0.01) increased in the laying hens fed the SBO-NPs. In ileum part, SBO-NPs also improved VH (p<0.01) and VSA (p<0.01) compared to other supplements. However, the inclusion of SBO and their NPs in the diets did not affect CD, VW, and VH:CD.

### Influence of dietary sweet basil oil-nanoparticle on cecal microbiota

The effect of dietary supplementation with SBO-NP on the taxonomic composition of the cecal microbiota is presented in Figure 1A, 1B. The cecal microbiota was dominated by the phylum *Bacillota* (Firmicutes). Minor phyla included *Bacteroidota*, *Actinobacteriota*, *and Pseudomonadota*, which together comprised 10%–20% of the community. The overall phylum-level profiles appeared broadly similar among the Con, SBO, and SBO-NP groups, although minor differences in the proportions of less abundant taxa were observed (Figure 1A). At the genus level, a diverse range of microorganisms was found, with *Lactobacillus* and *Clostridia* genera such as *Faecalibacterium*, *Ruminococcus*, *Roseburia*, and *Blautia*, which produce short-chain fatty acids (SCFA), being dominant. Although some variation in the relative abundance of specific genera was observed among individual samples, no major shift in the overall microbial profile was evident across treatments (Figure 1B).

Alpha diversity of the bacterial communities was assessed using the Chao1 and Shannon indices to evaluate richness and evenness. As demonstrated in Figure 2A, the Con sample showed consistent richness around 78 to 80. Both SBO and SBO-NP exhibited slightly higher median values (~83 to 85), suggesting an expansion of rare taxa. The SBO exhibited the greatest interindividual variation, indicating differential responses among birds to this diet. Regarding the Shannon index, the Con microbiota had a median value of approximately 3.4. The SBO-NPs displayed a modest increase (~3.5 to 3.55) with narrow variance. The SBO showed a wider range (3.2 to 3.7), reflecting greater heterogeneity among individuals (Figure 2B). However, all experimental diets did not affect microbial alpha diversity.

Beta diversity based on PCoA, used to assess separation of the microbial communities among the 3 groups (Figure 3). The Con formed a tight cluster, indicating low interindividual variation. SBO-NP overlapped with both the Con and SBO groups, showing moderate dispersion. The SBO exhibited the widest spread, suggesting high heterogeneity among birds receiving diet. However, PERMANOVA indicated that the overall microbial community structure was not significantly different among treatments (F = 0.997, R^2^ = 0.142, p = 0.465).

As shown in Figure 4, the bacterial populations in the cecum of laying hens fed the Con diet showed *Clostridioides difficile* and *Dorea formicigenerans* as the dominant microbial community. *Faecalibacterium prausnitzii*, *Lactobacillus helveticus, Intestinibacillus* sp., and *Blautia argi* were found in moderate to low levels. In addition, *Staphylococcus aureus*, *Enterococcus faecium*, and *Vescimonas fastidiosa* were found in low levels, and *Escherichia coli* was presented in some samples. The bacterial populations in laying hens fed the SBO diet had the same dominant microbial community as the Con diet. *Lactobacillus* and *Faecalibacterium* were found in many samples, but in low quantities; the overall distribution was quite diverse. However, *E. coli* and *Salmonella enterica* were found in some samples (Figure 4B). The heatmap of the SBO-NPs group revealed that *M. gemmiger*, *C. difficile, and D. formicigenerans* were the most prominent, whereas *M. gnavus, L. helveticus*, and *F. prausnitzii* exhibited moderate signals over a narrower range (Figure 4C).

### Influence of dietary sweet basil oil-nanoparticle on egg production and egg quality

The effect of dietary supplementation with SBO-NP on laying performance is illustrated in Table 5. No significant difference was found in ADFI and average effective weight (AEW) among the experimental groups. Remarkably, laying hens receiving SBO-NPs diet had the highest egg production at 81.14%, followed by the SBO diet at 76.95%, and the Con diet at 64.13%, respectively (p = 0.001). However, the FCR showed no difference.

As shown in Table 6, the effect of dietary supplementation with SBO-NP on egg quality, which was presented into two periods: 5 and 10 wk of the experiment. It was found that the experimental diets had no effect on shell hardness, shell thickness, albumen height, yolk color, yolk index, and HU at the first period. In 10 wk, the results showed a similarity trend. Except, eggshell thickness tended to increase in the Con diet supplemented with both forms of SBO (p = 0.089). The HU ranged from 80 to 82, with no difference among the experimental diets (p>0.05).

## DISCUSSION

The present study evaluated the potential of nanoencapsulated SBO as a phytogenic feed additive for laying hens. The estragole (77%) present in SBO can function as a nonenzymatic antioxidant. Regarding toxicological concerns, the additive incorporated into the diet at 500 ppm, as a level comparable to previously reported that dietary supplementation with basil essential oil at 500 mg/kg feed improved growth performance and meat quality in broiler chickens [12]. Nevertheless, further studies are needed to confirm the long-term safety of estragole-rich basil oil in laying hens.

The increased antioxidant capacity observed in the present study may be attributable to the bioactive compounds in SBO as act non-enzymatic antioxidants and function synergistically with enzymatic antioxidants to form a stronger antioxidant defense system. The SBO had a relatively high antioxidant activity, which is consistent with the study by Romano et al. [33]. The mechanism of its antioxidant activity can be explained as estragole reducing free radicals through electron transfer, while linalool has a hydroxyl group (-OH) which can donate hydrogen, making it a hydrogen donor molecule [34]. These represent the second line defense, helping to maintain antioxidant status in animal body [35]. SBO-NPs enhanced the CAT activity in the liver and T-AOC in both organs. Previous research has demonstrated that CS NPs for the delivery of alpha-lipoic acid alleviate the effects of heat stress in rabbits by enhancing CAT activity and T-AOC in serum [36]. Supplemental SBO-NP reduced MDA when compared to the Con. This observation agrees with El-Nekeety et al. [13] who found that basil oil reduced MDA in the liver and kidneys of mice. A mixture of phytogenic antioxidants reduced MDA levels and regulated the expression of antioxidant-related genes in heat-stressed breeder hens [37]. However, factors influencing antioxidant capacity in poultry include breed, environment, nutritional management practices and disease status. The selected tissue should also be representative of antioxidant status and reflect local oxidative stress associated with nutrient utilization [38].

Exogenous dietary antioxidants can regulate the concentration of reactive oxygen species (ROS), which cause destruction of the small intestine [35]. This study demonstrates that laying hens fed a diet containing SBO-NPs improved VH increased VSA and reduced CD than laying hens fed a diet containing SBO. This interpretation is supported by the reduced intestinal MDA levels as mentioned earlier, demonstrating lower lipid peroxidation and oxidative damage, which may have contributed to the improved intestinal morphology. Intestinal structural changes, including taller villi, a higher VH:CD ratio, and shorter CD, are indicators for a greater absorptive surface area, reduced epithelial cell turnover, and enhanced mucosal stability [39]. Our findings are in agreement with Basharat et al. [40], who clearly suggested that additives NPs improved jejunum morphology and reduced feed conversion ratio in broilers. Another possibility for improved intestinal integrity may be attributed to the antioxidant and anti-inflammatory properties of the alginate-CS wall material [41,42]. In fact, a structurally healthy intestine enables animals to utilize nutrients from feed more efficiently for productivity, while also functioning as a barrier against pathogens.

The balance of gut microbiota is influenced by several factors, including the rearing system, density, management practices, feed composition, housing conditions, hygiene, temperature, humidity, infections, and genetics [43]. The present microbiome results indicated that the cecal microbiota remained broadly similar among the Con, SBO, and SBO-NPs groups at the phylum and genus levels. The specific microbial groups showed changes, whereas the overall microbiota remained largely unchanged, reflecting that dietary supplementation has a relatively limited effect on core microbiota. This finding is consistent with the alpha diversity and beta diversity results, which indicated limited effects on microbial richness and diversity, with no clear separation of the overall microbial community structure among the treatment groups. Previous studies have shown that phytogenic extracts promoted intestinal microbial composition and metabolic function in chickens [44]. The SBO group was associated with fiber-degrading *Bacillus*-related bacteria, while SBO-NP group was associated with lactic acid and SCFA-producing genera such as *Lactobacillus, Enterococcus* and *Megasphaera*. In fact, SCFA serve as an energy source for maintaining intestinal health and productive performance in poultry, with butyrate being of particular significance [8]. However, the detection of *Enterobacteriaceae* and *Salmonella* at low levels may be attributed to the presence of estragole, linalool, and 1,8-cineole, which can disrupt cell membrane structures, leading to cytoplasm coagulation, lipids and protein denaturation. Similar results were stated by Ilić et al. [45], basil essential oil had a broad-spectrum antimicrobial activity against both gram-positive and gram-negative bacteria (i.e., *S. aureus*, *E. coli*, and *Proteus vulgaris*). This is supported by Beier et al. [46]. Nevertheless, experimental diets did not markedly alter the overall cecal microbiota structure. A possible explanation is that the bioactive compounds in SBO were delivered at the proximal intestine via the action of NP, leaving an insufficient available dose of SBO at the distal gut. Consequently, its effect on microbial community may have been limited. Further studies incorporating quantitative differential abundance analysis and microbial functional assessment are needed to clarify the biological significance of these changes.

All parameters as mentioned earlier are directly linked to hen’s productivity. No differences were observed in ADFI and AEW and FCR in this study. Similarly, encapsulated *Lavandula angustifolia* essential oil in alginate had no effect on FI or mortality [47]. However, it clearly impacted egg production. Administering SBO-NPs increased HD (%) by more than 26.52% and 6.53% compared to the Con and SBO groups. The greater egg production may be related to the well-intestinal morphology observed in the treated hens, supporting more efficient nutrient absorption. Whereas the increase in antioxidant capacity may contribute to improving the antioxidant defenses. Another possibility, phytogenic additives are involved in enhancing feed palatability and stimulating digestive enzymes, which influence digestibility [48]. This is confirmed by Gao et al. [49], who reported oregano essential oil did not affect ADFI while improving egg productivity in late-phase laying hens. In addition, the positive effect on productivity may be linked to antioxidant properties, which can protect the reproductive system from free radical damage in laying hens [50]. This study found no significant difference in internal egg quality; however, a trend toward increased eggshell thickness, which may have been influenced by additives. According to this study, *Ocimum sanctum* supplementation at 1 and 2 g/kg. did not affect overall egg quality [51]. This result implies that the mechanisms involving internal egg quality traits may not be sufficient to detect significant differences. Nevertheless, the tendency for greater eggshell thickness may indicate that essential oils facilitate calcium absorption and eggshell formation [52].

Our findings clearly demonstrate that SBO-NP are superior to SBO in antioxidant capacity and intestinal morphology. This greater efficacy may be explained by NP facilitating the release of active compounds in SBO in the proximal intestine, enhancing their availability and biological effects. However, the controlled release of the NPs in the distal intestine may have been limited, resulting in no effect on the gut microbiome composition. The possible controlled release mechanism can be explained as follows: the alginate-CS complex is resistant to gastric acid and enzymatic degradation due to electrostatic complexation. The SBO-NP begin to break down under alkaline conditions. Structurally, NP with a larger surface area facilitate the release of more core material, thereby enhancing their efficacy in the lower part of the GI tract [53]. Similar results were stated by Zhao et al. [54] who found that alginate-CS capsules remained protein in simulated gastric fluid and exhibited rapid release in simulated intestinal fluid.

## CONCLUSION

Laying hens fed a diet supplemented with SBO-NP exhibited increased egg production efficiency without affecting egg quality. The SBO-NP improved VH and VSA in all three parts of the small intestine. SBO-NP also increased SOD activity in the intestine and liver, while reducing MDA levels by 52% to 53% compared to the Con group. A comparative analysis demonstrated that SBO-NP are superior to SBO in terms of antioxidant capacity and intestinal morphology. No differences were observed in the alpha and beta diversity of the gut microbiota under the present conditions. However, the overall response of the laying hens suggests that SBO-NP have the potential to be used as a promising feed additive in layer production systems. Furthermore, the knowledge gained from this study can be further developed into commercial animal feed supplements.

## Figures and Tables

**Figure 1:**
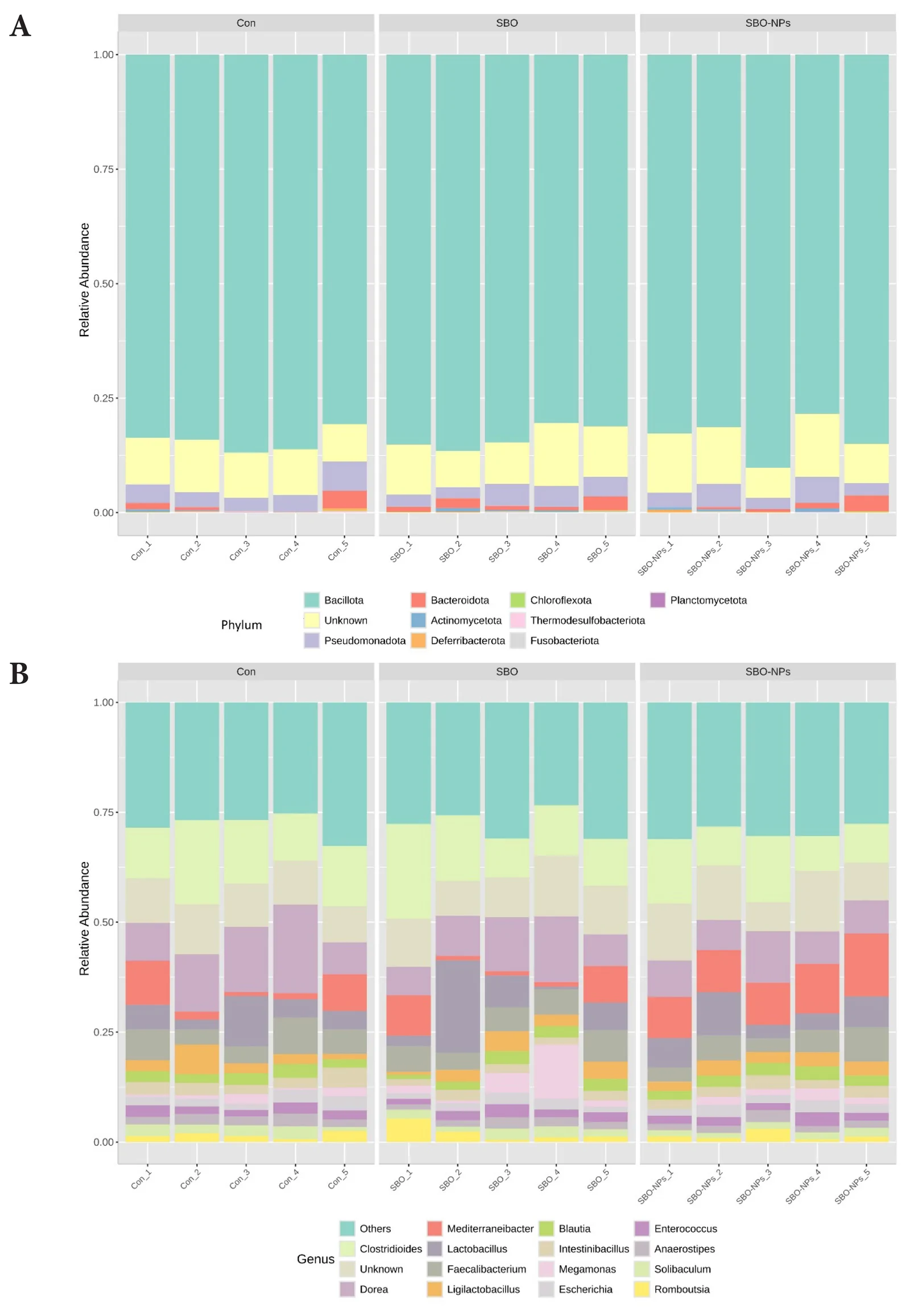
Taxonomic compositions of cecal microbiota in laying hens at 34 wk of age at the phylum (A), and genus (B) levels.

**Figure 2:**
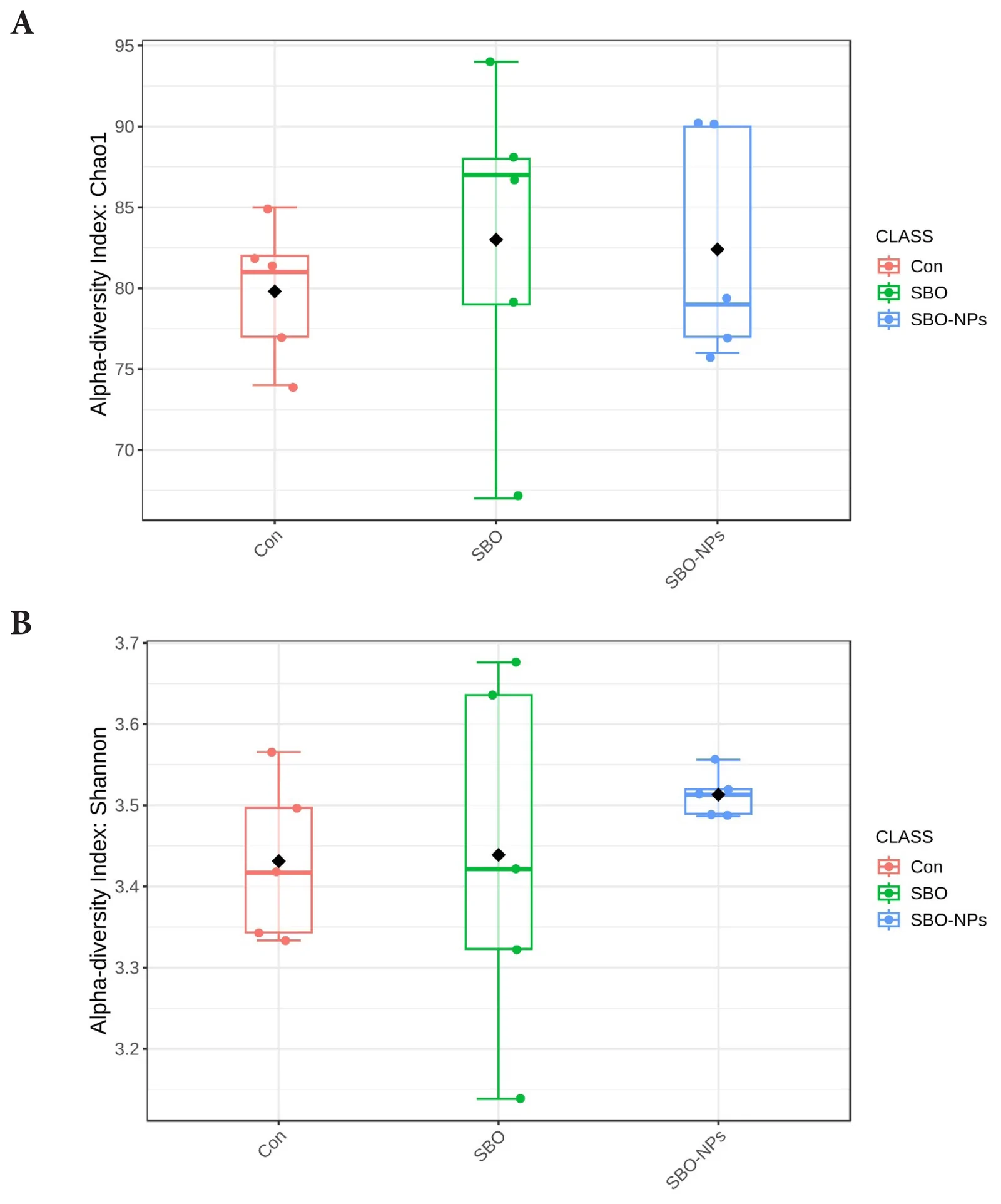
Alpha diversity of gut microbiota in laying hens at 34 wk of age. (A) Chao1, p = 0.74 and (B) Shannon index, p = 0.62. SBO-NPs, sweet basil oil nanoparticles; Con, basal diet, sweet basil oil at 500 ppm, and sweet basil oil-nanoparticles at 500 ppm.

**Figure 3:**
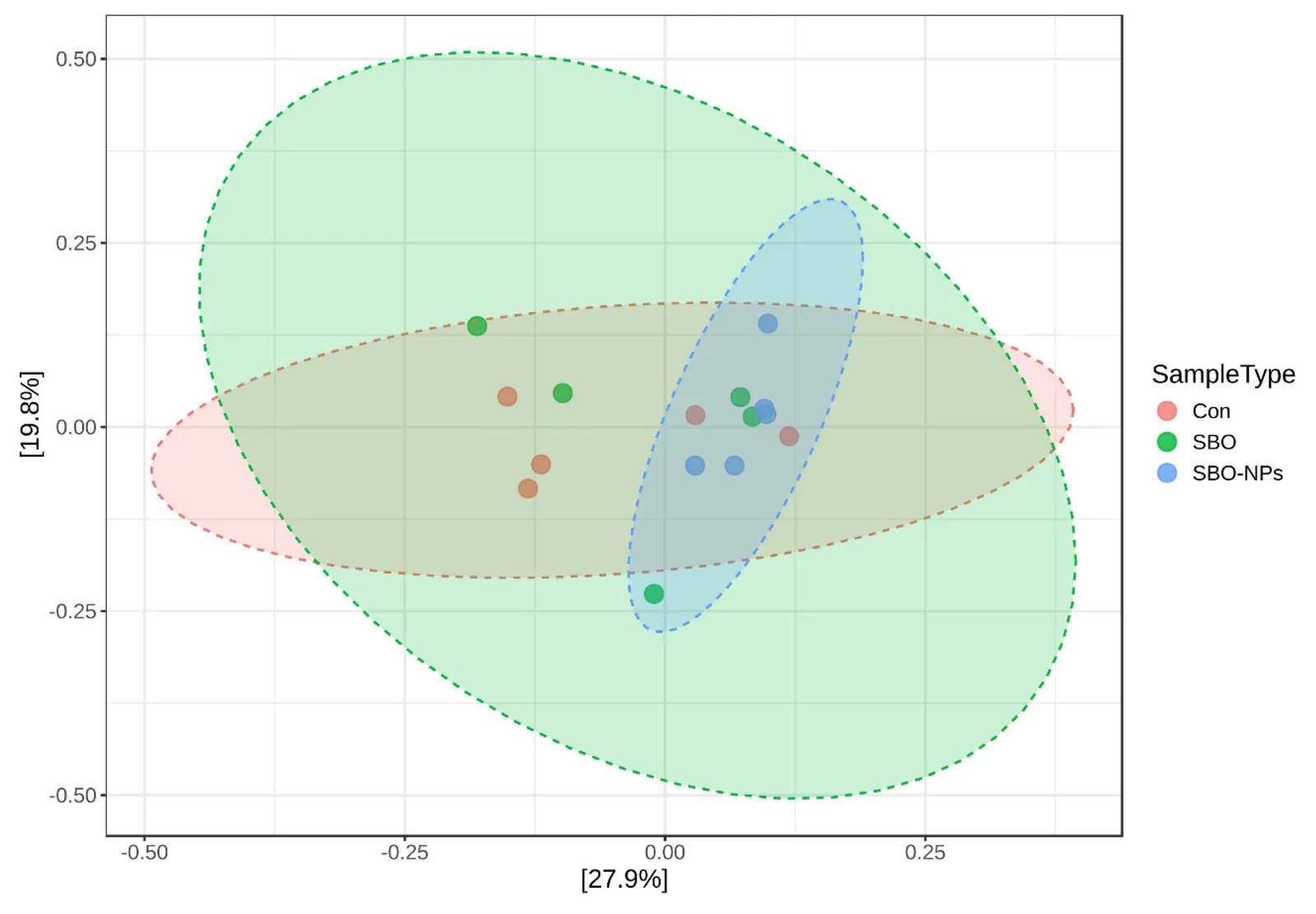
Beta diversity of gut microbiota in laying hens at 34 wk of age based on Bray–Curtis dissimilarity. Principal coordinates analysis (PCoA): R-squared: 0.1425; p-value: 0.465. Con, basal diet; SBO, sweet basil oil at 500 ppm; and SBO-NPs, sweet basil oil nanoparticles at 500 ppm.

**Figure 4:**
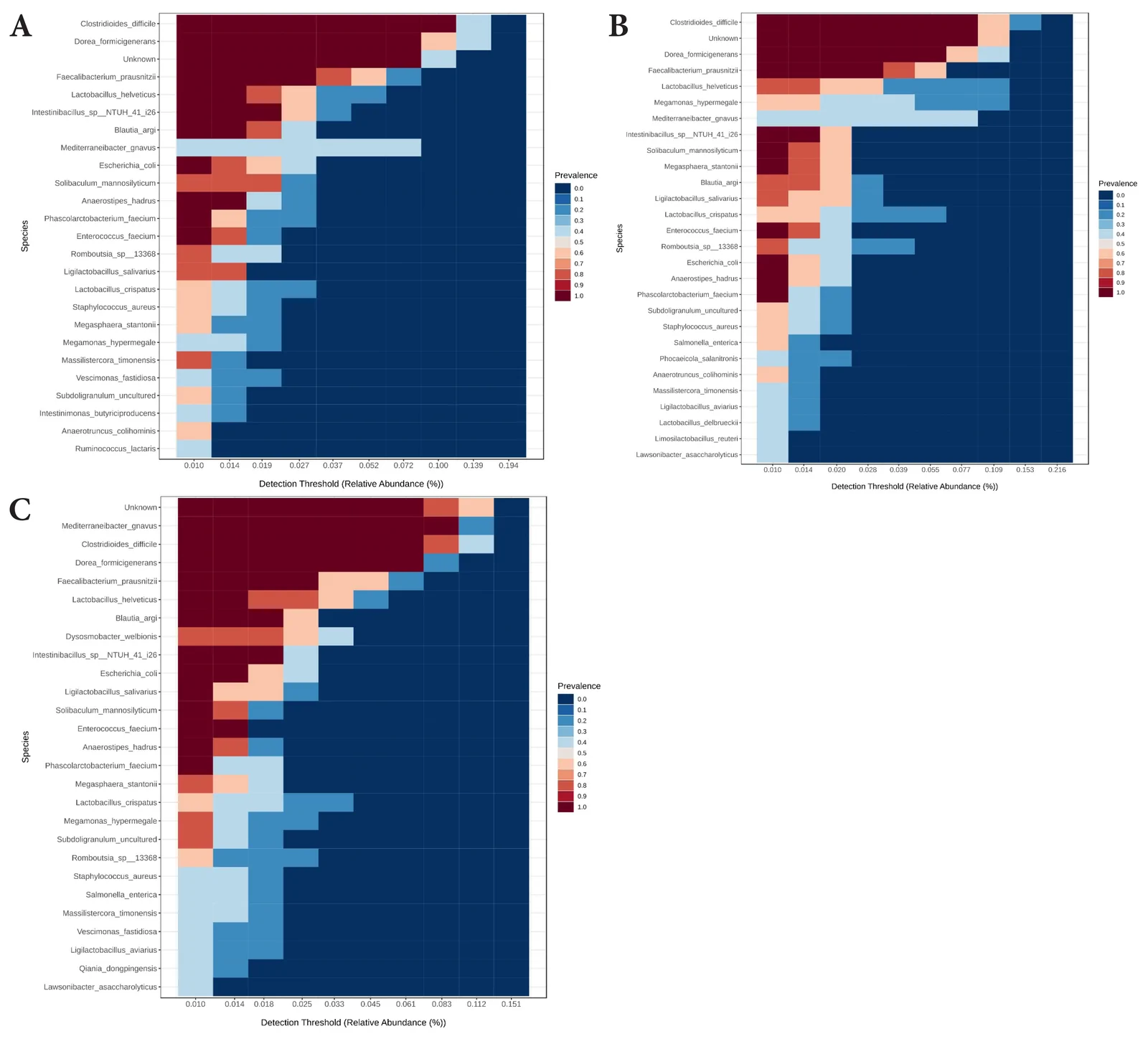
Relative abundance of microbial taxa representing gut microbiome. (A) Con: basal diet, (B) sweet basil oil at 500 ppm, and (C) sweet basil oil-nanoparticles at 500 ppm.

**Table 1:** Ingredients and chemical composition of the experimental diet

Ingredients (% as-fed)	Content
Corn	61.0
Soybean meal, 44% crude protein (CP)	24.0
Fish meal, 50% CP	3.0
Calcium carbonate	4.0
Limestone	3.0
Salt	0.2
Dicalcium phosphate, 18% P	3.0
DL-methionine	0.3
L-lysine	0.5
L-threonine	0.2
Choline chloride 60%	0.5
Premix-layer hen1)	0.3
Calculated nutrients (% as-fed)	
ME (MJ/kg)	12.45
CP	17.5
Crude fiber (CF)	2.0
Ether extract (EE)	5.5
Calcium (Ca)	3.7
Total phosphorus	0.9
Available phosphorus	0.2
Methionine	0.5
Lysine	1.2
Methionine+cystine	0.8
Tryptophan	0.1
Threonine	0.7
Sodium	0.2
Chloride	0.3
Analyzed nutrients (% as-fed)	
CP	17.8
CF	2.5
EE	5.2
Ca	3.8
Phosphorus	1.0

1) Provided per kilogram of diet: vitamin A 9,900 IU, vitamin D3 4,000 IU, vitamin E 25 IU, vitamin K 32.5 IU, vitamin B1 2 mg, vitamin B2 6 mg, vitamin B6 4 mg, vitamin B12 0.024 mg, biotin 0.2 mg, pantothenic acid 10 mg, nicotinamide 35 mg, folacin 1 mg, choline 360 mg, Fe 80 mg, Cu 10 mg, Mn 100 mg, Zn 100 mg, 1.1 mg, Se 0.3 mg, and methionine 1.5 g.

**Table 2:** Bioactive constituents in sweet basil oil

Peak	Name	Retention time	Area	%Area
1	(1R)-2,6,6-Trimethylbicyclo[3.1.1] hept-2-ene	8.622	515,723	0.11
2	β-Myrcene	10	2,126,013	0.45
3	Cyclohexene, 1-methyl-5-(1-methylethenyl)-	11.496	1,300,227	0.28
4	Eucalyptol	11.65	5,433,068	1.15
5	β-Ocimene	11.891	11,214,556	2.38
6	Linalool	13.777	36,531,970	7.75
7	(+)-2-Bornanone	16.063	2,447,352	0.52
8	Estragole	17.985	365,582,673	77.51
9	2,6-Octadienal, 3,7-dimethyl-, (E)-	19.625	840,474	0.18
10	(+)-Borneol acetate	21.752	809,222	0.17
11	Caryophyllene	27.884	1,233,308	0.26
12	trans-α-Bergamotene	28.146	13,661,903	2.9
13	α-Guaiene	28.409	914,902	0.19
14	cis- β-Farnesene	28.646	639,201	0.14
15	1,4,7,-Cycloundecatriene, 1,5,9,9-tetramethyl-,Z,Z,Z-	29.295	2,210,798	0.47
16	Germacrene D	30.371	3,514,196	0.75
17	β-Bisabolene	31.038	584,299	0.12
18	gamma.-Muurolene	31.602	2,072,888	0.44
19	Cyclohexene, 4-[(1E)-1,5-dimethyl-1,4-hexadien-1-yl]-1-methyl-	32.346	3,689,304	0.78
20	Others			3.45

**Table 3:** Effects of dietary sweet basil oil-nanoparticles (SBO-NP) on hepatic and intestinal antioxidant status of laying hens at 34 wk of age

Item	Treatment1)			SEM	p-value

	Con	SBO	SBO-NP		
Duodenum					
T-AOC (μM Trolox equivalent)	0.41^c^	0.69^b^	0.79^a^	0.113	0.001
SOD (U/mL)	134.64	137.42	139.72	1.469	0.556
CAT (U/mL)	5.04	5.14	5.21	0.050	0.439
GSH-Px (U/mL)	40.16	39.79	40.77	0.287	0.843
MDA (nmol/mL)	6.27^a^	4.05^b^	2.95^c^	0.977	0.001
Liver					
T-AOC (μM Trolox equivalent)	0.33^b^	0.40^a^	0.40^a^	0.022	0.022
SOD (U/mL)	116.37	119.70	122.06	1.652	0.301
CAT (U/mL)	3.56^b^	3.69^b^	3.96^a^	0.116	0.001
GSH-Px (U/mL)	37.57	37.69	38.18	0.185	0.384
MDA (nmol/mL)	5.07^a^	4.02^b^	2.45^c^	0.761	0.001

Means were calculated using 5 replicates per treatment (n = 5).

1) Con: basal diet, SBO: basal diet with sweet basil oil at 500 ppm, and SBO-NPs: basal diet with SBO nanoparticles at 500 ppm, respectively.

a–c Within a row, means with no common superscript differ significantly (p<0.05).

SEM, standard error of the mean; T-AOC, total antioxidant capacity; SOD, superoxide dismutase; CAT, catalase; GSH-Px, glutathione peroxidase; MDA, malondialdehyde.

**Table 4:** Effects of dietary sweet basil oil-nanoparticles (SBO-NP) on intestinal morphology of laying hens at 34 wk of age

Item	Treatments1)			SEM	p-value

	Con	SBO	SBO-NP		
Duodenum					
Villus height (VH, μm)	1,201.51^b^	1,228.93^b^	1,310.70^a^	32.795	0.001
Crypt depth (CD, μm)	64.46^b^	63.13^b^	70.19^a^	2.166	0.001
Villus width (VW, μm)	170.17^b^	178.17^ab^	190.62^a^	5.950	0.010
Villus height to crypt depth ratio (VH:CD)	5.81	6.02	5.93	0.061	0.865
Villus surface area (VSA, mm^2^)	2.44^b^	2.43^b^	2.91^a^	0.157	0.001
Jejunum					
VH (μm)	938.53^c^	1,031.60^b^	1,110.59^a^	49.726	0.001
CD (μm)	59.62^b^	63.05^a^	64.91^a^	1.549	0.002
VW (μm)	181.02	174.56	173.75	2.298	0.590
VH:CD	5.09^b^	5.66^a^	5.79^a^	0.214	0.039
VSA (mm^2^)	1.78^c^	2.05^b^	2.27^a^	0.142	0.001
Ileum					
VH (μm)	924.80^c^	985.30^b^	1,070.08^a^	42.133	0.001
CD (μm)	60.59	62.45	64.55	1.147	0.138
VW (μm)	158.98	171.35	165.88	3.578	0.209
VH:CD	5.35	5.44	5.80	0.137	0.155
VSA (mm^2^)	1.79^c^	1.96^b^	2.18^a^	0.114	0.001

Means were calculated using 5 replicates per treatment (n = 5).

1) Con: basal diet, SBO: basal diet with sweet basil oil at 500 ppm, and SBO-NP: basal diet with SBO nanoparticles at 500 ppm, respectively.

a–c Within a row, means with no common superscript differ significantly (p<0.05).

SEM, standard error of the mean; VH, villus height; CD, crypt depth; VW, villus width; VH:CD, villus height to crypt depth ratio; VSA, villus surface area.

**Table 5:** Effects of dietary sweet basil oil-nanoparticles (SBO-NP) on production performance of laying hens from 25 to 34 wk of age

Item	Treatments1)			SEM	p-value

	Con	SBO	SBO-NP		
Initial weight (g)	1,604.27	1,610.40	1,609.60	1.925	0.978
ADFI (g/d/hen)	117.82	117.82	120.99	1.056	0.357
Hen day production (%)	64.13^b^	76.95^a^	81.14^a^	5.116	0.001
AEW (g)	51.72	52.47	51.44	0.308	0.627
FCR (feed to egg)	3.81	3.38	3.29	0.145	0.106

Means were calculated using 5 replicates per treatment (n = 5).

1) Con: basal diet, SBO: basal diet with sweet basil oil at 500 ppm, and SBO-NPs: basal diet with SBO nanoparticles at 500 ppm, respectively.

a,b Within a row, means with no common superscript differ significantly (p<0.05).

SEM, standard error of the mean; ADFI, average daily feed intake; AEW, average egg weight; FCR, feed conversion ratio.

**Table 6:** Effects of dietary sweet basil oil-nanoparticles (SBO-NP) on egg quality of laying hens at 29 and 34 wk of age

Item	Treatments1)			SEM	p-value

	Con	SBO	SBO-NP		
5 wk of experiment					
Shell strength (N)	43.19	44.13	42.60	0.45	0.812
Shell thickness (mm)	0.37	0.38	0.38	0.00	0.870
Albumen height (mm)	6.44	6.68	6.48	0.07	0.819
Yolk color	8.08	7.60	7.97	0.14	0.123
Yolk index	0.41	0.41	0.42	0.00	0.425
Haugh unit	81.19	82.62	80.61	0.60	0.827
10 wk of experiment					
Shell strength (N)	47.18	48.67	46.10	0.75	0.126
Shell thickness (mm)	0.39	0.41	0.41	0.01	0.089
Albumen height (mm)	6.47	6.46	6.57	0.04	0.932
Yolk color	7.99	7.88	8.14	0.07	0.560
Yolk index	0.41	0.42	0.41	0.00	0.787
Haugh unit	80.35	80.79	81.49	0.33	0.863

Means were calculated using 5 replicates per treatment (30 eggs/replicate).

1) Con: basal diet, SBO: basal diet with sweet basil oil at 500 ppm, and SBO-NPs: basal diet with SBO nanoparticles at 500 ppm, respectively.

SEM, standard error of the mean.

## Data Availability

Upon reasonable request, the datasets of this study can be available from the corresponding author.

## References

[1] WATT Global Media (c2025). Poultry trends. EGG CONSUMPTION: eggs’ accessibility and affordability drive consumption [Internet].

[2] Sun Y, Li Z, Yan M, Zhao H, He Z, Zhu M (2024). Responses of intestinal antioxidant capacity, morphology, barrier function, immunity, and microbial diversity to chlorogenic acid in late-peak laying hens. Animals.

[3] Xu M, Liu L, Fan Z (2025). Effect of different dietary oil sources on the performance, egg quality and antioxidant capacity during the late laying period. Poult Sci.

[4] Oladokun S, Adewole DI (2022). Biomarkers of heat stress and mechanism of heat stress response in Avian species: current insights and future perspectives from poultry science. J Therm Biol.

[5] Deng W, Dong XF, Tong JM, Zhang Q (2012). The probiotic Bacillus licheniformis ameliorates heat stress-induced impairment of egg production, gut morphology, and intestinal mucosal immunity in laying hens. Poult Sci.

[6] Wen C, Yan W, Mai C (2021). Joint contributions of the gut microbiota and host genetics to feed efficiency in chickens. Microbiome.

[7] Shahid MAH, Jha R, Mishra B (2026). Changes in the gut microbiome, metabolic pathways, and intestinal gene expression during the peak, mid, and decline egg production phases in laying hens. Poult Sci.

[8] Hussain G, Obianwuna UE, Wen C (2025). Dietary broccoli stem-and-leaf meal in laying hens: effects on production performance, egg quality, antioxidant status, immune function, and gut microbiota composition. Poult Sci.

[9] López-Hernández M, Criollo-Nuñez J, Beltran JI, Sandoval-Aldana A (2024). Chemical composition and antioxidant activity of ‘Nufar’ basil (Ocimum basilicum L.) essential oil from three municipalities of Tolima, Colombia. Food Chem Adv.

[10] Fachriyah E, Wibawa PJ, Awaliyah A (2020). Antibacterial activity of basil oil (Ocimum basilicum L) and basil oil nanoemulsion. J Phys Conf Ser.

[11] Thuekeaw S, Buwjoom T, Maneewan B, Nuengjamnong C (2023). Porous alginate microcapsule-containing sweet basil oil (Ocimum basilicum) generated by gas foaming with ionic gelation as an efficient antioxidant delivery in stimulated poultry gastrointestinal tract. ACS Food Sci Technol.

[12] Vlaicu PA, Untea AE, Panaite TD, Saracila M, Turcu RP, Dumitru M (2023). Effect of basil, thyme and sage essential oils as phytogenic feed additives on production performances, meat quality and intestinal microbiota in broiler chickens. Agriculture.

[13] El-Nekeety AA, Hassan ME, Hassan RR (2021). Nanoencapsulation of basil essential oil alleviates the oxidative stress, genotoxicity and DNA damage in rats exposed to biosynthesized iron nanoparticles. Heliyon.

[14] Elmeligy EH, Attallah ST, Sallam EA, Mohammed LS (2025). Impact of Yucca extract and basil oil supplementation on carcass characteristics, quality of meat, and the cecal microbiota in broiler chickens. J Adv Vet Res.

[15] Manzari-Tavakoli A, Babajani A, Tavakoli MM, Safaeinejad F (2024). Integrating natural compounds and nanoparticle-based drug delivery systems: a novel strategy for enhanced efficacy and selectivity in cancer therapy. Cancer Med.

[16] Hosseintabar-Ghasemabad B, Ondrašovičová S, Kvan OV (2025). Applications and impacts of nanotechnology in poultry nutrition. Discov Appl Sci.

[17] Chin S, Moniruzzaman M, Smirnova E (2024). Green metal nanotechnology in monogastric animal health: current trends and future prospects: a review. Anim Biosci.

[18] Hashem NM, Hosny NS, El-Desoky N (2023). Alginate nanoencapsulated synbiotic composite of pomegranate peel phytogenics and multi-probiotic species as a potential feed additive: physicochemical, antioxidant, and antimicrobial activities. Animals.

[19] Ibrahim D, Abdelfattah-Hassan A, Badawi M (2021). Thymol nanoemulsion promoted broiler chicken’s growth, gastrointestinal barrier and bacterial community and conferred protection against Salmonella Typhimurium. Sci Rep.

[20] Maity S, Mukhopadhyay P, Kundu PP, Chakraborti AS (2017). Alginate coated chitosan core-shell nanoparticles for efficient oral delivery of naringenin in diabetic animals: an in vitro and in vivo approach. Carbohydr Polym.

[21] Yousefdoost S, Samadi F, Jafari SM, Ramezanpour SS, Hassani S, Ganji F (2019). Application of nanoencapsulated silymarin to improve its antioxidant and hepatoprotective activities against carbon tetrachloride-induced oxidative stress in broiler chickens. Livest Sci.

[22] Amiri N, Afsharmanesh M, Meimandipour A (2024). Effects of free and nanoencapsulated garlic essential oil on growth performance and immune functions in broiler chickens. Anim Sci J.

[23] Adil S, Banday MT, Sheikh IU (2023). Improvement in the gut microstructure and nutrient digestibility by dietary inclusion of nanoencapsulated lavender essential oil in broiler chicken. Ind J Anim Health.

[24] Qasem A, Assaggaf H, Mrabti HN (2023). Determination of chemical composition and investigation of biological activities of Ocimum basilicum L. Molecules.

[25] Caponio GR, Miolla R, Vacca M, Difonzo G, De Angelis M (2024). Wine lees as functional ingredient to produce biscuits fortified with polyphenols and dietary fibre. LWT-Food Sci Technol.

[26] Brand-Williams W, Cuvelier ME, Berset C (1995). Use of a free radical method to evaluate antioxidant activity. LWT Food Sci Technol.

[27] Thuekeaw S, Angkanaporn K, Nuengjamnong C (2022). Microencapsulated basil oil (Ocimum basilicum Linn.) enhances growth performance, intestinal morphology, and antioxidant capacity of broiler chickens in the tropics. Anim Biosci.

[28] National Research Council (1994). Nutrient requirements of poultry.

[29] Mierlita D, Dărăban S, Macri A, Pop IM (2025). Effect of dietary wine lees on oxidative stress, egg quality and yolk lipid stability in laying hens fed with a diet rich in n-3 polyunsaturated fats. Vet Anim Sci.

[30] Habig WH, Pabst MJ, Jakoby WB (1974). Glutathione S-transferases: the first enzymatic step in mercapturic acid formation. J Biol Chem.

[31] Beers RF, Sizer IW (1952). A spectrophotometric method for measuring the breakdown of hydrogen peroxide by catalase. J Biol Chem.

[32] Mierlita D, Teuşdea AC, Matei M, Pascal C, Simeanu D, Pop IM (2024). Effect of dietary incorporation of hemp seeds alone or with dried fruit pomace on laying hens’ performance and on lipid composition and oxidation status of egg yolks. Animals.

[33] Romano R, De Luca L, Aiello A (2022). Basil (Ocimum basilicum L.) leaves as a source of bioactive compounds. Foods.

[34] Ahmed AF, Attia FAK, Liu Z, Li C, Wei J, Kang W (2019). Antioxidant activity and total phenolic content of essential oils and extracts of sweet basil (Ocimum basilicum L.) plants. Food Sci Hum Wellness.

[35] Oke OE, Akosile OA, Oni AI (2024). Oxidative stress in poultry production. Poult Sci.

[36] El-Raghi AA, Al Doghaither HA, Al-Ghafari AB (2025). Enhancement of growth performance and health status in heat-stressed growing rabbits using alpha-lipoic acid-loaded chitosan nanoparticles. Anim Biosci.

[37] Pasri P, Rakngam S, Gérard N, Mermillod P, Khempaka S (2024). Synthetic and phytogenic antioxidants improve productive performance, antioxidant activity, gene expression, and offspring quality in breeder hens subjected to heat stress. Poult Sci.

[38] Maiorka A, Santin E, Dahlke F, Boleli IC, Furlan RL (2003). Posthatching water and feed deprivation affect the gastrointestinal tract and intestinal mucosa development of broiler chicks. J Appl Poult Res.

[39] El Sabry MI, Yalcin S (2023). Factors influencing the development of gastrointestinal tract and nutrient transporters’ function during the embryonic life of chickens: a review. J Anim Physiol Anim Nutr.

[40] Basharat S, Tahir SK, Majeed KA (2023). Effects of iron oxide nanoparticle supplementation on the growth performance, serum metabolites, meat quality, and jejunal basal morphology in broilers. Animals (Basel).

[41] Lukova P, Kokova V, Baldzhieva A (2024). Alginate from Ericaria crinita possesses antioxidant activity and attenuates systemic inflammation via downregulation of pro-inflammatory cytokines. Mar Drugs.

[42] Wang Z, Yan Y, Zhang Z (2024). Effect of chitosan and its water-soluble derivatives on antioxidant activity. Polymers.

[43] Shen X, Huang W, Zhan S (2025). Bamboo leaf flavonoids counteract lipopolysaccharide-induced gut dysfunction in aging laying hens through microbiota and metabolic reprogramming. Poult Sci.

[44] Zhu N, Wang J, Yu L, Zhang Q, Chen K, Liu B (2019). Modulation of growth performance and intestinal microbiota in chickens fed plant extracts or virginiamycin. Front Microbiol.

[45] Ilić ZS, Milenković L, Šunić L (2021). Efficiency of basil essential oil antimicrobial agents under different shading treatments and harvest times. Agronomy.

[46] Beier RC, Byrd JA, Kubena LF (2014). Evaluation of linalool, a natural antimicrobial and insecticidal essential oil from basil: effects on poultry. Poult Sci.

[47] Adaszyńska-Skwirzyńska M, Zych S, Grabowska M (2026). Effects of encapsulated Lavandula angustifolia essential oil in alginate hydrogel capsules as feed additives to enhance the performance and health of broiler chickens and its in vitro antibacterial activity against multidrug-resistant Escherichia coli strains. Poult Sci.

[48] Stevanović ZD, Bošnjak-Neumüller J, Pajić-Lijaković I, Raj J, Vasiljević M (2018). Essential oils as feed additives: future perspectives. Molecules.

[49] Gao F, Zhang L, Li H (2022). Dietary oregano essential oil supplementation influences production performance and gut microbiota in late-phase laying hens fed wheat-based diets. Animals.

[50] Radwan Nadia L, Hassan RA, Qota EM, Fayek HM (2008). Effect of natural antioxidant on oxidative stability of eggs and productive and reproductive performance of laying hens. Int J Poult Sci.

[51] Kirubakaran A, Narahari D, Ezhil Valavan T, Sathish Kumar A (2011). Effects of flaxseed, sardines, pearl millet, and holy basil leaves on production traits of layers and fatty acid composition of egg yolks. Poult Sci.

[52] Darmawan A, Öztürk E, Güngör E, Özlü Ş, Jayanegara A (2024). Effects of essential oils on egg production and feed efficiency as influenced by laying hen breed: a meta-analysis. Vet World.

[53] Castro-Valenzuela BE, Franco-Molina MA, Rodríguez-Padilla C (2025). Nanoparticles as an alternative treatment for bovine mastitis: a review. Anim Biosci.

[54] Zhao B, Alonso NF, Miras J (2024). Triggered protein release from calcium alginate/chitosan gastro-resistant capsules. Colloids Surf A Physicochem Eng Asp.

